# Clinical evaluation of atlas-based auto-segmentation in breast and nodal radiotherapy

**DOI:** 10.1259/bjr.20230040

**Published:** 2023-07-25

**Authors:** Camarie Welgemoed, Emiliano Spezi, Pippa Riddle, Mark J Gooding, Dorothy Gujral, Ruth McLauchlan, Eric O Aboagye

**Affiliations:** 1 Radiotherapy Department, Imperial College Healthcare NHS Trust, Charing Cross Hospital, London, United Kingdom; 2 Department of Surgery and Cancer, Imperial College London, Hammersmith Campus, London, United Kingdom; 3 School of Engineering, Cardiff University, Cardiff, United Kingdom; 4 Mirada Science Group, Oxford, United Kingdom; 5 Department of Radiation Physics & Radiobiology, Imperial College Healthcare NHS Trust, Charing Cross Hospital, London, United Kingdom; 6 Blackett Laboratory, Imperial College London, South Kensington Campus, London, United Kingdom

## Abstract

**Objectives:**

Accurate contouring of anatomical structures allows for high-precision radiotherapy planning, targeting the dose at treatment volumes and avoiding organs at risk. Manual contouring is time-consuming with significant user variability, whereas auto-segmentation (AS) has proven efficiency benefits but requires editing before treatment planning. This study investigated whether atlas-based AS (ABAS) accuracy improves with template atlas group size and character-specific atlas and test case selection.

**Methods and materials:**

One clinician retrospectively contoured the breast, nodes, lung, heart, and brachial plexus on 100 CT scans, adhering to peer-reviewed guidelines. Atlases were clustered in group sizes, treatment positions, chest wall separations, and ASs created with Mirada software. The similarity of ASs compared to reference contours was described by the Jaccard similarity coefficient (JSC) and centroid distance variance (CDV).

**Results:**

Across group sizes, for all structures combined, the mean JSC was 0.6 (*SD* 0.3, *p* = .999). Across atlas-specific groups, 0.6 (*SD* 0.3, *p* = 1.000). The correlation between JSC and structure volume was weak in both scenarios (adjusted *R* ^2^−0.007 and 0.185).

Mean CDV was similar across groups but varied up to 1.2 cm for specific structures.

**Conclusions:**

Character-specific atlas groups and test case selection did not improve accuracy outcomes. High-quality ASs were obtained from groups containing as few as ten atlases, subsequently simplifying the application of ABAS. CDV measures indicating auto-segmentation variations on the x, y, and z axes can be utilised to decide on the clinical relevance of variations and reduce AS editing.

**Advances in knowledge:**

High-quality ABASs can be obtained from as few as ten template atlases.

Atlas and test case selection do not improve AS accuracy.

Unlike well-known quantitative similarity indices, volume displacement metrics provide information on the location of segmentation variations, helping assessment of the clinical relevance of variations and reducing clinician editing. Volume displacement metrics combined with the qualitative measure of clinician assessment could reduce user variability.

## Introduction

Breast cancer is the most common cancer in the world.^1^ 63% of breast cancer patients receive radiotherapy as part of their primary treatment.^2^ Adjuvant loco-regional radiotherapy significantly reduces recurrence and mortality in breast and nodal cancer.^3–5^ Outcomes from a multicentre phase 3 trial strongly recommended lymph node (LN) contouring and three-dimensional (3-D) radiotherapy planning.^6^ Efficient 3-D planning requires dose conformity to treatment volumes and improves treatment outcomes. Likewise, minimising the dose delivered to organs at risk (OAR) reduces side effects.^7^

Accurate contouring is, therefore, an integral part of high-precision radiotherapy. Manual contouring, however, is time-consuming and subject to substantial inter- and intra-observer variations.^8–10^ Although contouring guidelines have proven accuracy improvements, other factors, including training differences, image quality, human error, clinician variability, interpretation, radiological input and clinical experience levels, contribute to variability.^11,12^

While commercial atlas-based auto-segmentation (ABAS) lacks accuracy, it has improved workflow efficiency, as editing of auto-segmentation (AS) is more efficient than contouring from scratch.^6,13–18^ The models typically provide institutions with template atlases because it can be expensive, time-consuming, and not practical in a busy radiotherapy workflow to create expert/template atlases. These commercial atlases do not necessarily represent local, national, or international contouring standards and, as a result, may require excessive and time-consuming modifications, potentially swaying RT departments from adopting AS as standard practice. However, the software allows the user to create and group template atlases (local previously contoured images). When creating new AS, the algorithm draws information from template atlases. Deformable image registration (DIR) brings the atlas images in precise spatial correspondence to the patient/test case image and deforms the atlas contours to the patient coordinates. Utilising a subset of atlases instead of the entire database optimises the search for suitable atlases, reduces DIR iteration, and increases computational speed.^19^ Successful AS solutions mutually rely on the number and quality of expert template atlases and DIR.

Most AS clinical validation studies have been performed on head and neck sites. ABAS is more challenging to implement in the abdomen and thorax because of anatomical variations between patients and organ movement within anatomical cavities.^19^ AS reports in breast radiotherapy often include only a selection of structures required during breast and regional nodal irradiation.^20–22^ Furthermore, the optimal number of atlases appears to be an overlooked research topic; most studies included 10–20 atlases per group.^20,23,24^ To improve AS accuracy, Rohlfing proposed selecting atlas templates more suitable for a specific patient from large databases.^25^ An ABAS study on brain magnetic resonance imaging (MRI) also reported improved accuracy when using optimum atlas selection and an increased number of selected templates. However, high accuracy has been achieved by fewer templates with appropriate anatomical variability. Wu concluded that different ROIs might require different atlas numbers.^26^ Theoretically, perfect atlas selection on an extensive database may lead to AS equivalent to expert manual outlines.^15^ Others reported that AS contours for the breast better represented the “true” volume than it did for the brachial plexus and supraclavicular nodes. Despite corrections, they also reported time-saving benefits and confirmed AS correction is required before progressing with planning.^15^ Based on dosimetric assessment results, a multi-institutional study recommended AS for the clinical setting to save time, despite the need for editing before planning.^6,27^

We hypothesised that ABAS outcomes could be enhanced by developing an atlas database for a clinical setting, and selecting atlas and test cases with specific characteristics when creating AS.

The study sought to find a clinic-friendly model to optimise AS and reduce clinician editing. Deep learning (DL) AS methods were not explored due to the belief that more data lead to better results.^28,29^ Our dataset comprised of 100 high-quality CT structure sets, suitable for creating categorical clustering using ABAS and avoiding test case duplication in atlas groups.

## Methods and materials

### Building the atlas library and quality assurance

**Figure 1.**
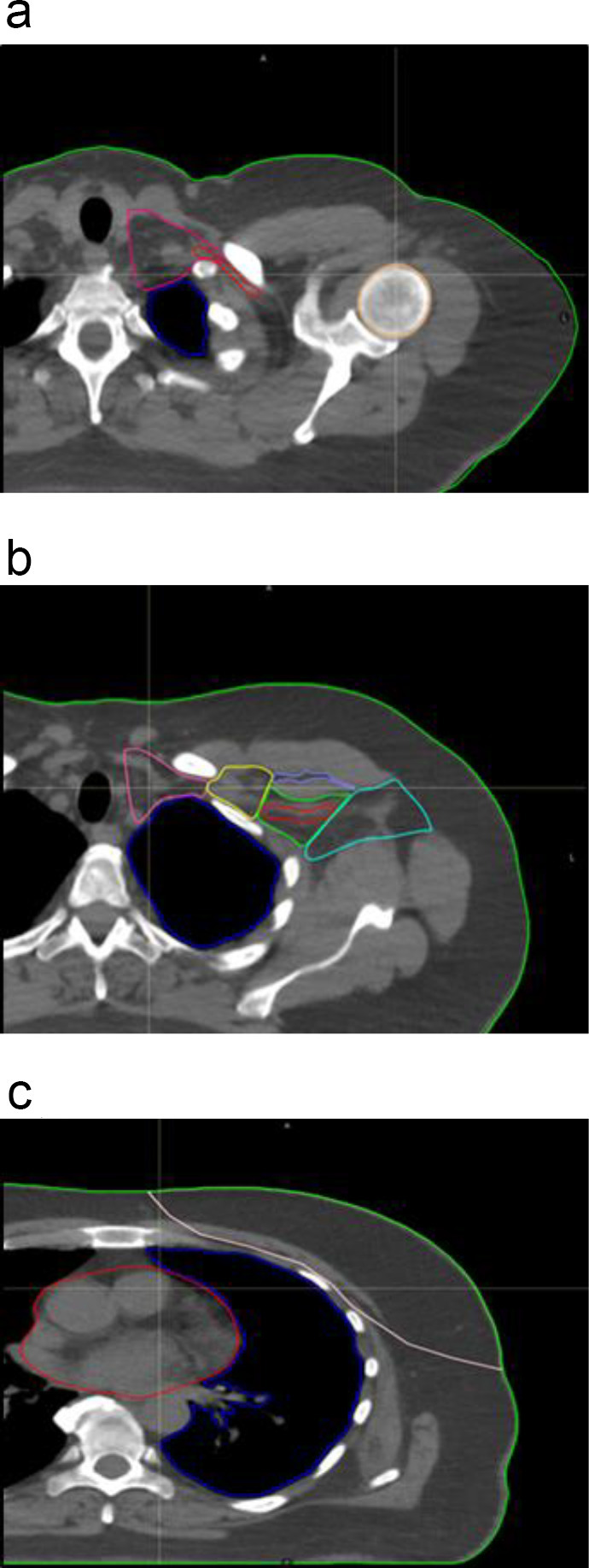
Typical CT axial slice images with manual segmentations of normal and nodal structures utilised during radiotherapy planning. a) demonstrates the level 4 nodes (pink), humeral head (orange), brachial plexus (red), and lung (blue). Figure 1b) level 4 - (pink), level 3 - (yellow), level 2 - (green), level 1 - (aquamarine), and inter-pectoral nodes (lilac), brachial plexus (red), and lung (blue). Figure 1c) lung (blue), heart (red), and breast clinical target volume (salmon).

Given that template atlas accuracy influences AS outcomes,^19^ we created a database of CT structure sets consisting of highly standardised contours. 100 radiotherapy planning CTs were randomly selected, and anatomical structures retrospectively contoured, utilising Prosoma, V.4. Oncology Systems Limited, UK. Contours included the breast, regional nodes, and organs at risk (Figure 1). We also combined individual nodal groups: levels 3 and 4, and levels 1, 2, 3, and 4 as indicated by clinical target volumes (CTV) utilised during radiotherapy planning.

The dataset was partitioned into atlases and test cases. Template atlases were clustered into atlas groups, and the test case images were used for AS, which were compared to the reference contours of the same CT image.

One expert clinician followed peer-reviewed guidelines to minimise inter-observer variability and ensure consistency. The European Society of Radiotherapy and Oncology (ESTRO), Radiation Therapy Oncology Group, and Hall guidelines were used for the breast and nodes, heart and brachial plexus, respectively.^30–32^ The same clinician and two independent consultant breast oncologists verified the contours for accuracy and to minimise intra-observer variability.

### Atlas Group-Selection

Pseudonymised CT structure sets were transferred to AS software (Mirada Workflow box 1.4, Mirada Medical Ltd., UK). By random selection, we created seven atlas groups consisting of 10, 20, 25, 30, 40, 45, and 50 heterogenous/mixed atlases. AS were created on ten test cases to determine if accuracy relates to the atlas group size. Different test case images were used on the groups with 25 and 45 atlases to improve robustness.

**Figure 2.**
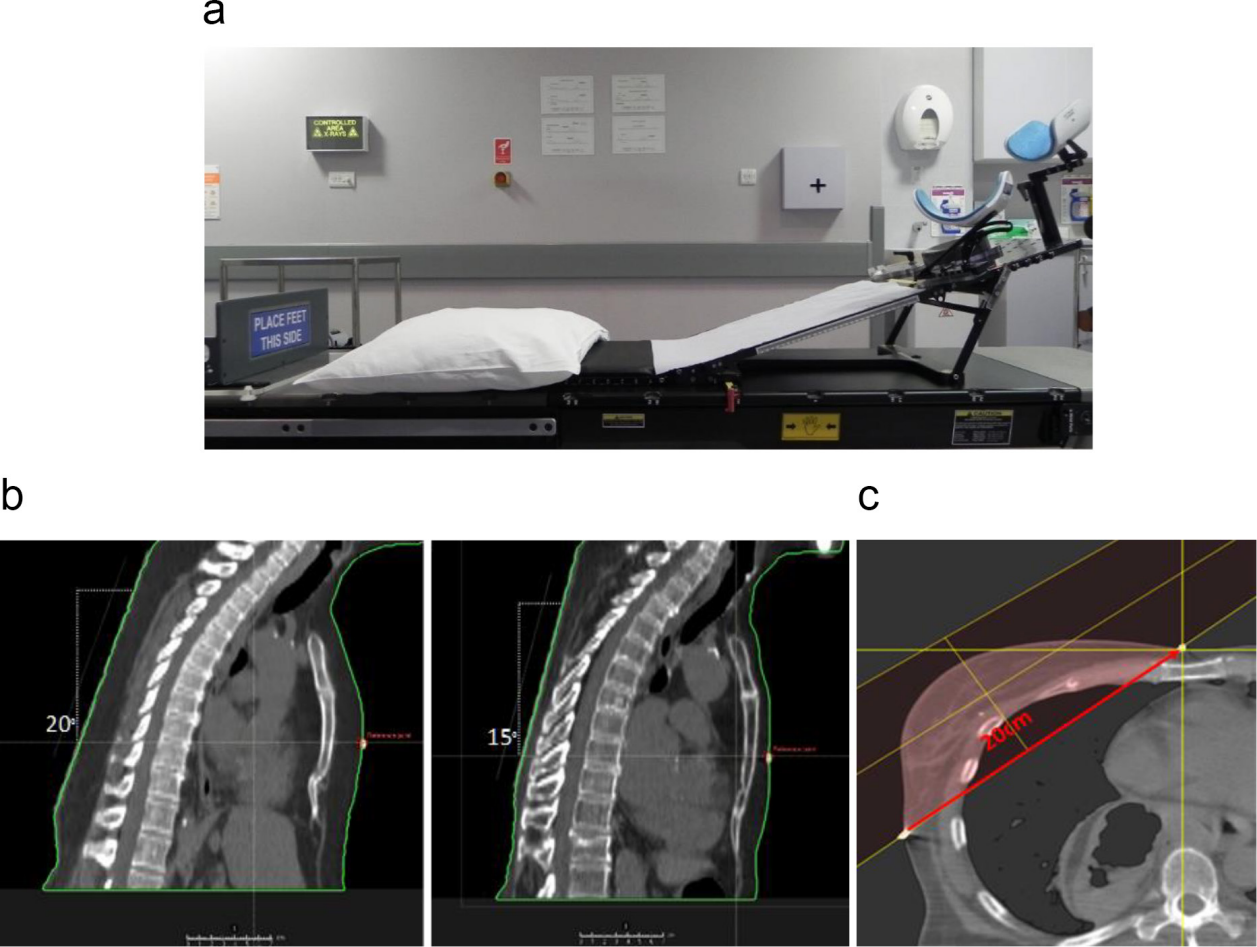
Atlases were ranked according to non-image-based information such as treatment position and chest wall separation to create character-specific atlas groups. a) demonstrates a breast board with an adjustable incline, 2b) a scan position with 20° incline (left) and 15° (right), and 2c) a chest wall separation as measured between two skin markers.

Descriptive file names facilitated offline atlas sampling, creating character-specific atlas groups, and preventing bias by avoiding duplicate template atlases. Atlases were ranked according to non-image-based information such as treatment position and chest wall separation (Figure 2). Treatment position groups included breast board incline variables of 15° and 20°. Chest wall (CW) separation was determined by the distance between medial and lateral CT markers. All atlases in the ‘Large’ group were of a large separation, scanned on 15° or 20° incline. The '20° Large' group included atlases with large separation, scanned at a 20° incline. Consequently, the similarity in atlas groups '20° Large' and '15° Small' surpassed that of the 'Large' and '15°' groups. Finally, we compared categorised test cases with categorised atlases, distinguishing between matching (MTC) and non-matching test cases (NMTC)

### Registration and fusion

We used the Workflow Box for atlas segmentation. Mirada uses rigid registration followed by DIR. The study utilised multi-atlas segmentation (MAS), where registration and deformation are repeated on several atlases, and the deformed field is fused into a consensus contour.^19,33,34^ The algorithm is a derivative of the Lucas-Kinade Optic Flow.^35,36^

### Analysis method

**Figure 3.**
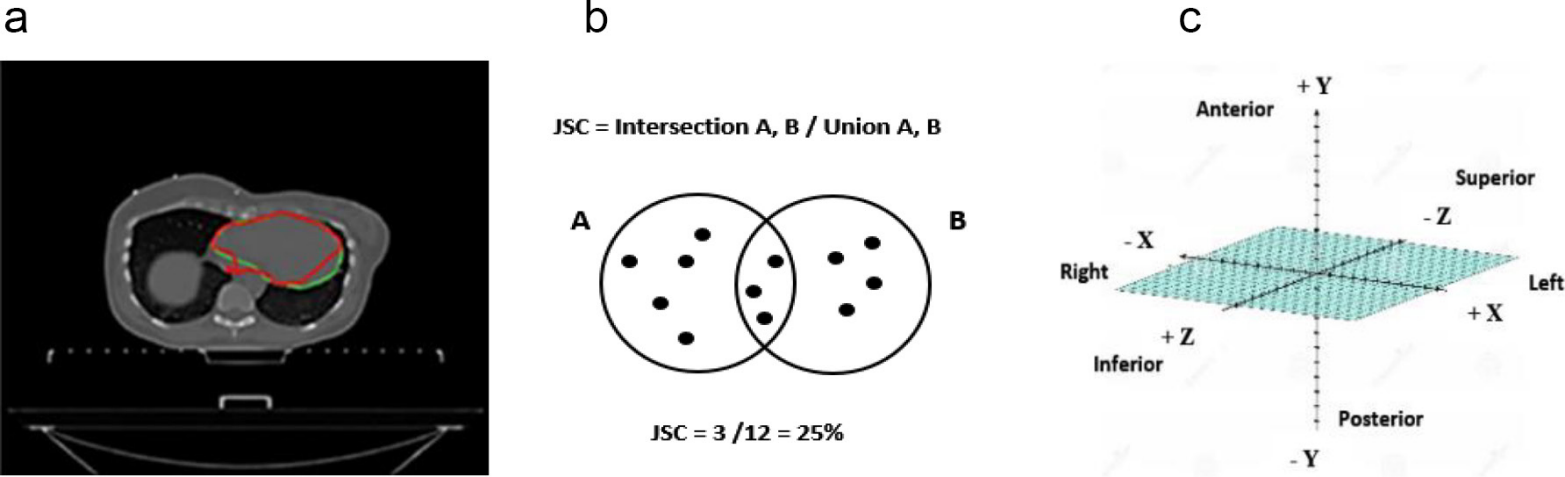
Auto-segmentation and reference image overlap measures. a) demonstrates the super-imposed auto-segmentation (red) and reference (green) contours of the heart, 3b) the Jaccard similarity coefficient (JSC) formula, and 3c) the 3D CT X, Y, and Z coordinate system and anatomical orientation that was utilised during calculation of centroid distance variants.

Figure 3a demonstrates a typical variation between an AS and the reference contour of the heart.

Since under- or over-contouring results in under- or over-treatment, we described the intersection over the union between structures with the Jaccard similarity coefficient (JSC)^37^ in Figure 3b. The metric ranges between 0 and 1; 1 indicates 100% overlap between structures, therefore, a high degree of AS efficacy.

Volume overlap metrics, like the JSC, do not necessarily reflect the clinical relevance of AS variations.^25,37,38^ Therefore, we determined volume displacement between reference contours and AS by calculating the centroid distance variants (CDV). Centroid distances were defined as the most medial, lateral, superior, inferior, anterior, and posterior extent of a structure to the structure centre, on the x, y, and z coordinate systems that relates to a CT scan axial slice (Figure 3c). CDV data for treatment structures with a JSC < 0.5 (inter-pectoral and internal mammary nodes), and the heart and lung were not analysed in this research; heart and lung structures are routinely successfully contoured by treatment planning systems. Both the JSC and CDV were calculated using the SPAARC software package.^39^

We analysed the impact of atlas numbers, atlas-specific groups, and test case selection on the effectiveness of automated segmentation, comparing JSC and CDV across groups. Positive CDV values indicated over-contouring, while negative values indicated under-contouring compared to reference contours. We also examined CDV means for specific structures to determine the clinical relevance of variations and clinician modifications. Additionally, we gathered data on the time required for creating reference contours and AS, without emphasising time savings.

### Statistical methods

Microsoft Excel 365, version 2019, was used to calculate descriptive statistics including the means, standard deviations (*SD*), contouring times, and *p*-values of JSC and CDV. One-way analysis of variance was used to compare the means between different groups and calculate R^2^; adjusted values were reported.

### Ethical considerations

The Health Research Authority and Research Governance Manager approved this retrospective data analysis study (REC reference 11/HRA/0379). Imperial College London sponsored the study.

## Results

The average AS and manual contouring time per case was 107 min (SD 23) and 4 min, respectively.

**Figure 4.**
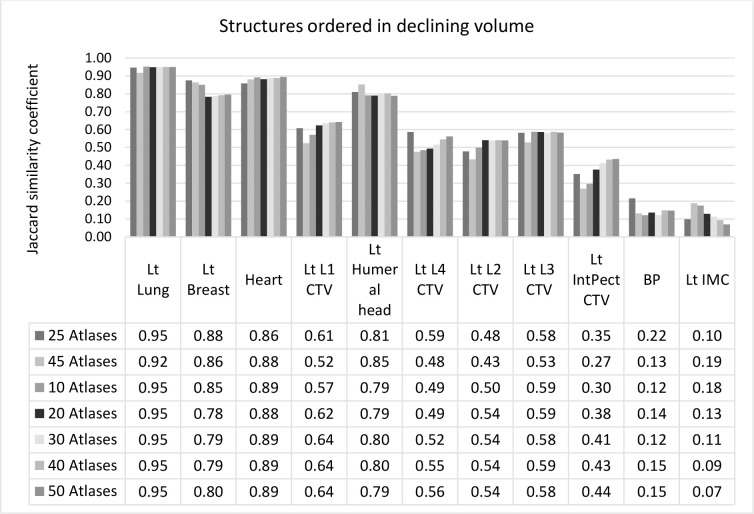
Volume overlap outcomes for different atlas group sizes demonstrates the Jaccard similarity coefficients (JSC) values, sorted by the size of structure volumes, across group sizes. Atlases in these groups were randomly selected and are heterogeneous.

We explored the association between the JSC and atlas group sizes (Figure 4). For all structures combined, the mean JSC was 0.6 (*SD* 0.3, *p* = .999) in groups consisting of 10, 20, 25, 30, 40, and 45 atlases, respectively. In the same atlas groups, the mean JSC for larger structures combined (breast, heart, lung, and level one nodes) measured 0.8 (*SD* 0.2, *p* = .999), and 0.4 (*SD* 0.2, *p* = .999) for smaller structures combined. The correlation between JSC and group sizes (*R* ^2^ = −0.20), and JSC and structure volume (*R* ^2^ = −0.007) was weak.

**Figure 5.**
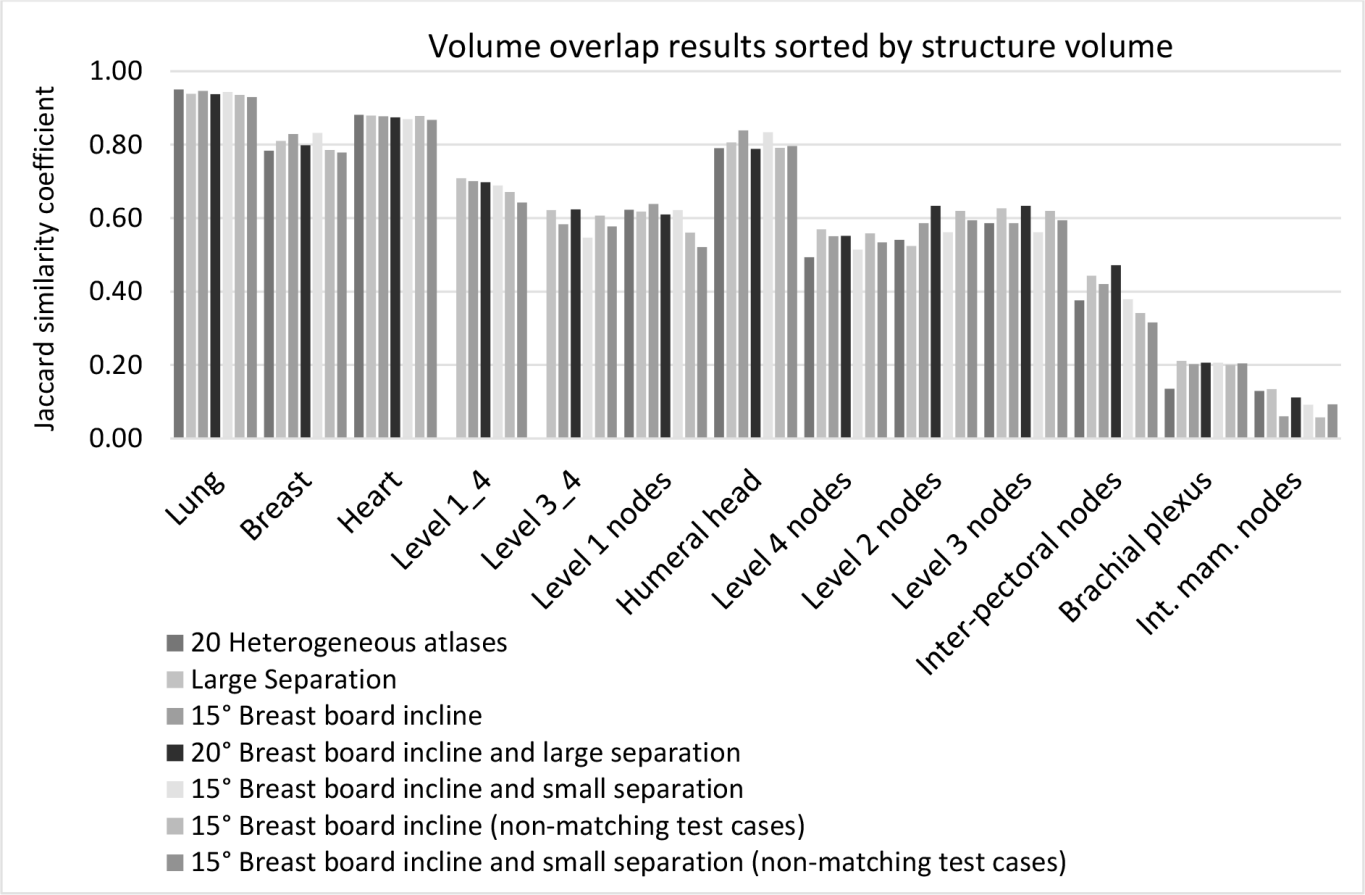
Volume overlap outcomes for character-specific atlas groups demonstrates JSC values sorted by the size of structure volumes across atlas-specific groups. The heterogeneous atlas group consisted of randomly selected atlases, not matching specific chest wall separation or breast board inclines. The “Large separation” group consisted of separations in the large range, scanned on either 20° or 15° inclines. All cases in “15° Breast board” group were scanned on a 15° Breast board but could be of a large or small separation. Cases in the ”20° Breast board incline and large separation” group were of a large separation and scanned on a 20° breast board. The non-matching test cases utilised in the last two groups: “15° Breast board incline”, and “15° Breast board incline and small separation”, did not match the atlas characteristics and, hence, were scanned on a 20° board and of a large chest wall separation. Apart from the “heterogeneous” group, all other test cases matched their atlas group characteristics.

We also explored the association between JSC and atlas-specific groups, based on different breast board inclines, chest wall separation, non-matching test cases, and heterogeneous/mixed atlases. Data for individual structures are demonstrated in Figure 5, ranging from the largest to lowest structure volumes. The mean JSC for all structures combined were 0.6 (*SD* 0.3, *p* = 1.000), larger structures 0.8 (*SD* 0.15, *p* = .999), and smaller structures 0.5 (*SD* 0.3, *p* = 1.000). The combined lymph node volumes, levels 1 to 4, and levels 3 to 4 were not included in this analysis. The correlation between JSC and atlas category (*R* ^2^ = 0.027), and) JSC and structure volume (*R* ^2^ = −0.185) was also weak.

CDV results for atlas groups (Large MTC, 15° NMTC, and Heterogeneous) and structures are shown in (Table 1). Including all the structures, the overall mean variant values were 0.1, 0, and 0.1 cm, respectively (SD 0.0). The mean CDV for specific structures, however, varied between 0 and 1.2 cm across atlas groups (Table 2).

**Table 1.** Centroid distance variants between reference and auto-segmentation 3D coordinate measures across atlas groups for specific structures

	Atlas groups	Min X	Max X	Min Y	Max Y	Min Z	Max Z
**BREAST**	*Large MTC*	0.2	0.1	−0.9	0.0	−0.2	−0.3
	*15° NMTC*	0.5	0.2	−0.7	0.0	0.1	−0.6
	*Heterogeneous group*.	−1.2	0.0	−0.7	−0.1	−1.2	−1.1
**LEVEL 1 NODES**	*Large MTC*	−0.1	0.4	−0.1	0.2	0.3	−0.2
	*15° NMTC*	−0.1	0.4	−0.8	0.0	0.1	−0.9
	*Heterogeneous group*.	−0.6	0.3	−1.2	0.1	0.8	0.8
**LEVEL 2 NODES**	*Large MTC*	0.1	0.6	−0.4	0.5	0.2	0.3
	*15° NMTC*	−0.4	1.4	−0.8	0.8	−0.1	2.0
	*Heterogeneous group*.	−0.4	0.2	−0.4	0.3	0.1	0.2
**LEVEL 3 NODES**	*Large MTC*	−0.1	0.6	−0.7	0.1	0.1	−0.4
	*15° NMTC*	−0.2	0.6	−1.0	0.2	0.0	−0.2
	*Heterogeneous group*.	−0.6	0.7	0.2	0.2	0.3	0.0
**LEVEL 4 NODES**	*Large MTC*	0.4	0.4	−0.6	0.2	0.2	−0.8
	*15° NMTC*	0.1	0.5	−0.7	0.2	0.4	−0.5
	*Heterogeneous group*.	−0.3	−0.5	−0.5	0.2	0.2	0.1
**LEVEL 3 & 4 NODES**	*Large MTC*	0.0	0.7	−0.7	0.3	0.3	−0.4
	*15° NMTC*	0.0	0.7	−1.0	0.3	0.4	−0.2
**LEVEL 1, 2, 3, & 4 NODES**	*Large MTC*	0.0	0.4	−0.4	0.1	0.6	0.1
	*15° NMTC*	0.0	0.4	−1.0	0.4	0.5	−0.1
**BRACHIAL PLEXUS**	*Large MTC*	−0.8	3.2	−0.6	0.9	−0.7	0.7
	*15° NMTC*	−1.4	2.3	−0.9	0.4	−0.2	0.5
	*Heterogeneous group*.	−3.5	0.0	−0.9	2.2	2.6	7.1
**HUMERAL HEAD**	*Large MTC*	−0.1	0.1	−0.1	0.0	0.4	0.0
	*15° NMTC*	−0.3	−0.1	0.0	−0.1	0.7	0.0
	*Heterogeneous group*.	0.0	−0.2	−0.2	−0.4	0.3	−0.1

This table demonstrates the minimum (Min) and maximum (Max) centroid distance variants on the x, y, and z coordinates across atlas groups. Negative variants refer to under-contouring and positive variants to over-contouring. The Large Matching test cases (MTC) atlas group/template consisted of atlases with a large separation, scanned on a 15° or 20° incline; the test cases matched the same atlas characteristics. The 15°non-matching test cases (NMTC) group consisted of atlases scanned on 15° incline and various separations; the test cases were scanned on 20° incline instead. The heterogeneous group consisted of randomly selected/mixed atlases, scanned on 15/20°.

**Table 2.** Mean centroid distance variants for the heterogeneous atlas group

HETEROGENEOUS ATLAS GROUP		REF Min X	Min X	REF Max X	Max X	REF Min Y	Min Y	REF Max Y	Max Y	REF Min Z	Min Z	Ref Max Z	Max Z
Variant location			Med		Lat		Post		Ant		Sup		Inf
**BREAST**	Mean	0.9	2.0	16.1	16.1	−2.1	−1.5	8.4	8.5	−9.4	−8.2	6.5	7.6
	SD	0.5	0.5	1.9	1.9	2.0	2.1	2.5	2.5	1.2	0.5	1.1	0.8
	Variant		−**1.2**		0.0		−**0.7**		−0.1		−1.2		−1.1
**LEVEL 1 NODES**	Mean	9.1	9.7	14.3	14.0	−3.3	−2.1	2.3	2.1	−11.0	−11.7	−3.8	−4.5
	SD	0.9	0.8	1.2	0.9	2.4	2.0	1.6	1.5	0.2	0.5	1.9	0.3
	Variant		−0.6		0.3		−**1.2**		0.1		0.8		0.8
**LEVEL 2 NODES**	Mean	6.4	6.8	10.6	10.4	−1.0	−0.6	3.7	3.4	−12.8	−12.9	−6.7	−6.9
	SD	0.6	0.8	1.1	0.8	1.0	1.1	1.3	1.5	0.5	0.5	0.3	0.3
	Variant		−0.4		0.2		−**0.4**		0.3		0.1		0.2
**LEVEL 3 NODES**	Mean	3.2	3.8	8.8	8.1	−0.1	−0.3	4.3	4.1	−12.8	−13.1	−9.4	−9.4
	SD		0.5	0.2	0.5	1.4	1.1	1.5	1.7	0.5	0.6	1.1	0.9
	Variant	0.4	−0.6		**0.7**		0.2		0.2		0.3		0.0
**LEVEL 4 NODES**	Mean	1.2	1.5	6.0	6.5	0.2	0.7	4.2	4.1	−13.2	−13.4	−10.6	−10.7
	SD	0.1	0.5	0.2	1.5	0.9	0.9	1.5	1.3	0.9	0.6	1.3	1.7
	Variant		−**0.3**		−**0.5**		−0.5		0.2		0.2		0.1
**BRACHIAL PLEXUS**	Mean	−0.2	3.3	10.7	10.7	−1.1	−0.3	4.2	2.0	−12.7	−15.3	−4.0	−11.1
	SD	2.7	0.6	3.0	0.7	1.5	1.4	1.8	0.9	8.9	0.8	10.2	0.6
	Variant		−3.5		0.0		−**0.9**		2.2		2.6		7.1
**HUMERAL HEAD**	Mean	11.1	11.1	15.7	15.9	−3.7	−3.5	1.1	1.5	−15.8	−16.1	−12.4	−12.3
	SD	0.7	0.7	0.9	0.7	1.2	1.2	1.8	1.6	1.0	0.5	0.2	0.3
	Variant		0.0		−0.2		−0.2		−0.4		0.3		−0.1

This table demonstrates the minimum (Min) and maximum (Max) centroid distance variants for reference image and auto-segmentations on the x, y, and z coordinates. Auto-segmentations for the breast, lymph nodes, brachial plexus, and humeral head have been created from the heterogeneous atlas group. The heterogeneous group consisted of randomly selected/mixed atlases, scanned on 15/20°.The variant location and anatomical orientation of the 3D x, y, and z coordinates have been included in the second row. Negative variants refer to under-contouring and positive variants to over-contouring. The values in bold indicate clinically relevant auto-segmentation variants, requiring correction before radiotherapy planning.

## Discussion

We developed a quality-assured CT database of contoured structures, routinely required during breast and nodal radiotherapy planning. The total manual contouring time for 100 cases, excluding checking and editing time, was 178 h. The CT structure sets were applied as training and test cases during ABAS.

While it is expected that JSC overlap results should increase with atlas group size, our research concluded that high-quality AS can be obtained from as few as ten locally generated CT-based atlases, which simplifies the application of ABAS in radiotherapy departments. Previous research in brain MRI images, based on a maximum of 13 atlases per group, suggested that multiple atlases outperformed the single atlas method.^26^ Although we did not explore the single atlas method, we explored between 10 and 50 consecutively acquired atlases per group and eliminated accuracy benefits from utilising larger than 10 atlas groups.

The JSC results for the heart, lung, breast, and humeral head were in the range (0.8, 0.9), compared to other studies that recorded the Sorenson Dice similarity coefficient (DSC) of 0.8,^21^ equalling 0.7 (JSC).^40^ The JSC for smaller structures (brachial plexus, interpectoral, and internal mammary nodes) ranged between (0.5, 0.6) across atlas groups. Ciardo et al reported comparable results for the lung, heart, brachial plexus, and supraclavicular nodes.^41^

Previously discussed atlas selection strategies in breast AS included thoracic circumference and laterality.^41^ This is the first research to stratify template atlases to different treatment positions and CW separation groups. The results demonstrated no significant JSC differences between character-specific and heterogeneous atlas groups, or between NMTC and MTC AS (Figure 5). Subsequently, patient CT scans do not have to match atlas features, and AS can be created from any combination of atlases, allowing for straightforward ABAS application in practice.

In the analyses of varying group sizes and atlas-specific groups, we confirmed a weak correlation between the JSC values and structure volume size. It is worth stating that the JSC metric is volume-related and lower JSC values do not necessarily correlate with more clinician editing; clinician editing is more likely to relate to breast tissue.^42^

This study included combined nodal CTVs consisting of levels 3 and 4, and levels 1, 2, 3, and 4 nodal groups, respectively. As the JSC results compare well with individually outlined nodal volumes (Figure 5), it may save time to outline combined CTVs instead of individual nodal groups for template atlases and avoid volume merging during radiotherapy planning.

Both the JSC and CDV metrics resulted in similar conformity differences across atlas groups and disprove the hypothesis that ABAS conformity outcomes can be improved by atlas or matching test case selection. The CDV, however, differed between atlas groups for individual structures, suggesting the application of different atlas groups/templates for individual structures to achieve optimal AS. As this will complicate application in clinical practice, it seems reasonable to utilise heterogeneous atlas groups and translate the knowledge on the location of variations into clinical relevance, reducing clinician editing. We identified 11 variant locations needing review before radiotherapy planning. The locations included the medial and posterior aspect of the breast, the posterior aspect of the level 1 and level 2 nodes, the lateral aspect of the level 3, the lateral and medial aspect of the level 4 nodes, and the medial, posterior and anterior aspect of the brachial plexus (Table 2).

Breast treatment planning frequently involves the placement of large tangential fields rather than 3-D conformal radiotherapy (3DCRT) to a planning target volume. We did not consider the correction of structures in the intersection between the breast and nodal fields. Due to low image contrast in tissue density between normal tissue, the breast parenchyma and level 1 nodal tissue, manual outline accuracy variations in the superior, posterior, and lateral aspects are common.^9^ Considering manual contouring variations, AS modifications are likely to be affected by similar issues and unlikely to significantly impact treatment plans.

Nodal treatment fields include combinations of level 4, levels 3 and 4, and levels 1, 2,3, and 4. Subsequently, field placements are determined by the medial and lateral border of the level 4 nodes, the lateral extent of the level 3 nodes, and the lateral extent of the level 1 nodes.

The mean AS variations on the medial (−0.3 cm) and lateral extent (−0.5 cm) of the level 4 nodes, and lateral extent of the level 3 nodes (0.7 cm) are likely to compromise PTV dose coverage which is clinically relevant and will require editing. However, when modifying the lateral extent of the level 3 nodes it is essential to avoid overlay between the treatment field and surgical clips and therefore, minimising radiation side effects.

The AS variations for level 1 (−1.2 cm) and 2 nodes (−0.4 cm) in the posterior direction (Table 1) are likely to affect the PTV dose coverage at depth which will be clinically relevant, and therefore, require correcting.

The level 2 nodes are located posterior to the interpectoral nodes, between the levels 3 and 1 nodes, and are never treated as a distinct nodal group. Medial and lateral field borders are unlikely to be affected by AS variations. Similarly, the humeral head anterior variation (mean 0.4 cm) does not define humeral head shielding during radiotherapy planning and would not require editing.

Manual contouring of the brachial plexus is problematic and varied; low tissue density and image contrast on CT scans make it challenging to identify landmark structures. Unsurprisingly, the CDV for the brachial plexus was greater than other structures. In 3DCRT, it is feasible to calculate the dose at the intersection between the first rib and clavicle, where the brachial plexus is likely to receive the highest dose. However, when planning IMRT and VMAT, contouring the entire brachial plexus is crucial. Using AS instead of manual contouring may be the preferred option to address these challenges.

Segmentations in this research were derived from a standardised and quality-assured “database” and may differ from template atlases that various clinicians have contoured. Relying on contouring by one person can be seen as both a study strength and weakness; inter-observer variability is minimised but may impact on the validity of drawn conclusions. However, contouring and reviewing clinicians attended ESTRO contouring guideline training to further minimise intra-observer variability and their experience levels were 10, 5, and 15 years, respectively.

Future research and development of this model require validation of more AS solutions and confirmation in a multi-institutional setting. We expect to publish our results on comparing this AS model with deep learning soon.

DL approaches show competitive results compared to ABAS in certain CTVs (*e.g.,* level 3 axillary and internal mammary nodes, heart) in terms of the dice similarity coefficient and 95% Hausdorff distance.^14^ However, reported values vary significantly, making consistent model comparisons difficult.^43^ Additionally, there is a need for standardised validation metrics to evaluate clinical acceptability and commissioning of AS models.^44^ The CDV measures used in this study indicate AS variations in the x, y, and z axes, highlighting the importance of modifications based on clinical relevance. The software solution used for data processing was validated against a digital phantom through IBSI international collaboration.^39,45^

## Conclusion

The study results disprove the hypothesis that ABAS conformity outcomes can be improved by atlas or matching test case selection. High-quality ABAS could be obtained from as few as ten template atlases. Furthermore, our findings verified that atlas selection, based on breast separation, treatment position, and MTC does not benefit AS efficacy and subsequently simplify ABAS application.

Despite the continuous development of AS methods, AS does not yet represent a perfect match to reference images. Our research demonstrated potential in applying the CDV as a metric to help reduce clinician editing in radiotherapy departments.
